# Recent Development and Environmental Applications of Nanocellulose-Based Membranes

**DOI:** 10.3390/membranes12030287

**Published:** 2022-03-01

**Authors:** Syafiqah Syazwani Jaffar, Suryani Saallah, Mailin Misson, Shafiquzzaman Siddiquee, Jumardi Roslan, Sariah Saalah, Wuled Lenggoro

**Affiliations:** 1 Biotechnology Research Institute, Universiti Malaysia Sabah, Kota Kinabalu 88400, Malaysia; syafiqahsyazwanii@gmail.com (S.S.J.); mailin@ums.edu.my (M.M.); shafiqpab@ums.edu.my (S.S.); 2 Faculty of Food Science and Nutrition, Universiti Malaysia Sabah, Kota Kinabalu 88400, Malaysia; jumardi@ums.edu.my; 3 Faculty of Engineering, Universiti Malaysia Sabah, Kota Kinabalu 88400, Malaysia; s_sariah@ums.edu.my; 4 Institute of Engineering, Tokyo University of Agriculture and Technology, Tokyo 184-8588, Japan; wuled@cc.tuat.ac.jp

**Keywords:** nanocellulose, membrane, water filtration, environmental remediation, adsorbent, photocatalyst

## Abstract

Extensive research and development in the production of nanocellulose production, a green, bio-based, and renewable biomaterial has paved the way for the development of advanced functional materials for a multitude of applications. From a membrane technology perspective, the exceptional mechanical strength, high crystallinity, tunable surface chemistry, and anti-fouling behavior of nanocellulose, manifested from its structural and nanodimensional properties are particularly attractive. Thus, an opportunity has emerged to exploit these features to develop nanocellulose-based membranes for environmental applications. This review provides insights into the prospect of nanocellulose as a matrix or as an additive to enhance membrane performance in water filtration, environmental remediation, and the development of pollutant sensors and energy devices, focusing on the most recent progress from 2017 to 2022. A brief overview of the strategies to tailor the nanocellulose surface chemistry for the effective removal of specific pollutants and nanocellulose-based membrane fabrication approaches are also presented. The major challenges and future directions associated with the environmental applications of nanocellulose-based membranes are put into perspective, with primary emphasis on advanced multifunctional membranes.

## 1. Introduction

**Figure 1:**
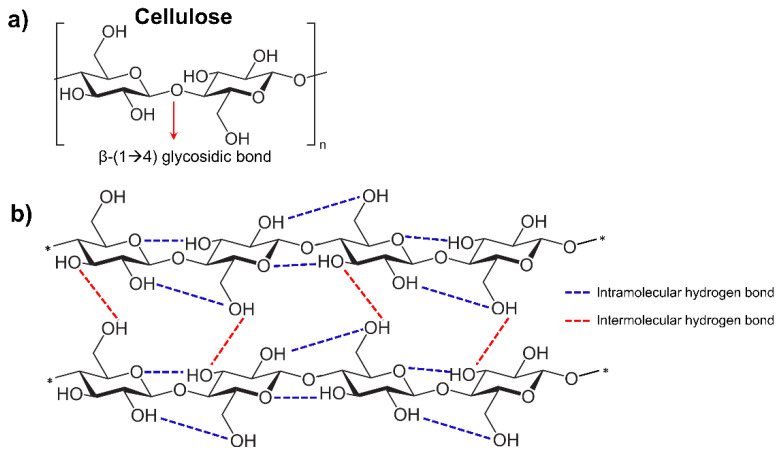
(**a**) Molecular structure of cellulose and (**b**) intramolecular (blue-dotted line) and intermolecular (red-dotted line) hydrogen bonding formation in cellulose.

Immense research and development have been made in green sustainable materials that exhibit outstanding characteristics and functionalities [1]. Cellulose is the most prevalent natural biopolymer on the earth and a major structural component of lignocellulosic biomass, accounting for up to 35–50% of the total biomass components [2]. It is composed of polysaccharides with long chains of β-D-glucopyranose units assembled by β-1,4 glycosidic bonds (Figure 1a) [3] and is characterized by the extensive network of intramolecular and intermolecular hydrogen bonding that provides the plant with rigidity and strength (Figure 1b). Due to its availability, renewability, biocompatibility, and biodegradability, cellulose is seen as a promising alternative for replacing petroleum-based polymers [4].

Nanotechnology involving cellulosic substrates has garnered enormous attention over the last few decades which affords nanocellulose, a new generation of nanomaterials with at least one dimension in the nanoscale. Owing to their physicochemical features, such as exceptional mechanical properties, reinforcing capabilities, low density, high stability, and their ability for surface modification, nanocellulose is considered a fascinating nature-based futuristic material [5]. Extraction of nanocellulose from a wide range of lignocellulosic biomass including woods (higher plants), agricultural by-products (e.g., wheat, rice, pineapple, banana, oil palm, etc.) [6,7,8,9], and bacterial cellulose [10] can be performed via physical, mechanical, and biological treatments [11,12,13]. Nanocellulose can be categorized into three types based on its source and extraction method: (1) cellulose nanocrystals (CNCs), rod-shaped with widths and lengths vary in the range of 5–70 nm and 100–250 nm, respectively; (2) cellulose nanofibers (CNFs) which are long entangled fibers with a diameter of <100 nm and a length of up to several microns; and (3) bacterial nanocellulose (BNC), which is produced using a bottom-up approach through bacterial synthesis [5].

Recent studies have made a significant contribution to the understanding of the many properties, applications, and functionalization of nanocellulose. To date, most reviews have been focused on a specific application such as adsorbents for heavy metals and dyes [14], photoremediation [15], carbon capture [16], and water desalination [17]. The crucial demand for a clean environment has led to nanocellulose-based membranes as an emerging separation technology in air and water filtrations. Membranes provide the following advantages over the traditional separation technologies such as precipitation, adsorption, and ion exchange: excellent separation efficiency, low energy consumption, low cost, simple operation, and no secondary pollution [18]. With a nano-scale dimension, nanocellulose has a large surface area and aspect ratio, which is advantageous for membrane development and modification. Nanocellulose has undergone a number of chemical modifications in order to increase its potential in membrane technology. These modifications can either change the nanocellulose’s chemistry or introduce functional groups to its surface [19]. Functional groups can be introduced via non-covalent surface functionalization, chemical surface modifications, and polymer grafting.

**Figure 2:**
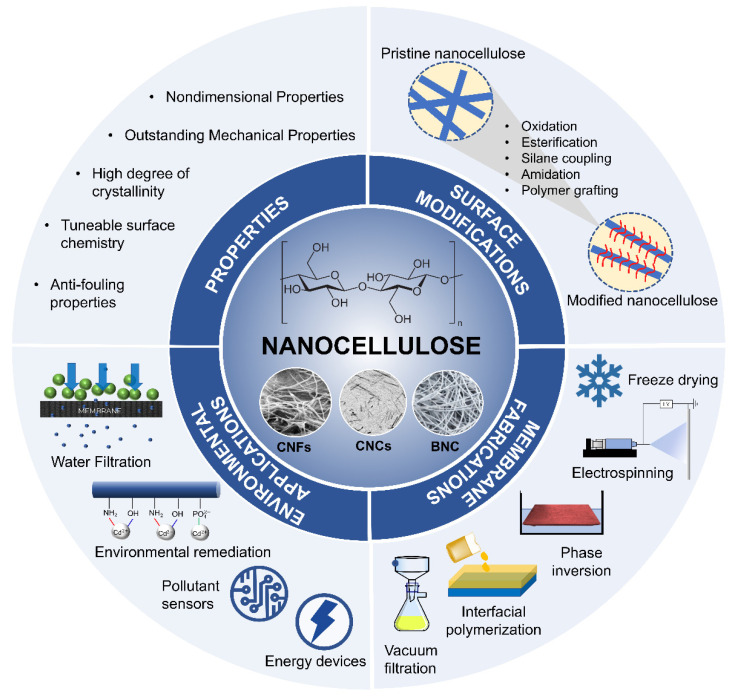
Overview of the highlights of the present review: desirable properties of nanocellulose, surface modification strategies, approaches for nanocellulose-based membrane fabrication and their environmental applications.

Rather than trying to review the already extensive literature on various sources and extraction methods of nanocellulose, this review focuses on recent advances in nanocellulose-based membranes, either as a membrane matrix or as an additive, modifier, and reinforcing agent for composite membranes with various surface modification strategies and fabrication methods. The main focus is on the recent progress of nanocellulose technology in environmental applications, particularly water filtration, environmental remediation, pollution sensors, and energy devices. The associated challenges with regard to the inherent properties and processing of nanocellulose are also addressed to gain insights into the technical feasibility of nanocellulose-based membranes for environmental applications. Finally, perspectives toward new directions for nanocellulose-based membranes advancements, notably in the context of multifunctional membranes are highlighted. The essence of the present review is depicted in Figure 2. Reference selection was conducted using the Google Scholar search function with the keywords: nanocellulose OR “cellulose nanofiber” OR “cellulose nanocrystal” AND “environmental application”, from the year 2017 until recently (2022).

## 2. Desirable Features of Nanocellulose from Membrane Technology Perspective

Nanocellulose is a sustainable nanomaterial that has received increasing attention in the last two decades owing to its desirable intrinsic cellulose properties such as eco-friendly nature, good biocompatibility, and low toxicity. The important properties of nanocellulose in the context of membrane technology are briefly discussed in this section, including nano-dimensional properties related to the high specific surface area, high aspect ratio, tunable porosity, outstanding reinforcing potential, high degree of crystallinity, tunable surface chemistry, and anti-fouling properties.

### 2.1. Nano-Dimensional Properties

The geometrical features of nanocelluloses which are defined by their length (L) and width (W) are mainly influenced by the source, extraction methods as well as processing conditions. The widths of CNCs range from 5 to 70 nm, with lengths ranging from 100 to 250 nm. CNFs have been described with a wider range of width (5 to 100 nm) and lengths of up to several microns have been reported, resulting in very high aspect ratios [20]. The interaction of nanocellulose with surrounding matrices is improved by increasing its specific surface area, which promotes high adsorption capacity and size-exclusion capabilities. Furthermore, nanocellulose’s high specific surface area facilitates surface functionalization via hydroxyl groups on the surface of cellulose fibrils, which attract pollutants. The specific surface area of nanocellulose has been reported to be between 50 and 200 to 200 m^2^/g. Nanocellulose in the form of aerogels has a substantially greater specific surface area (250–350 m^2^/g) with a much lower density (0.02 g/cm^3^) and a significant porosity of 98% [21].

In the context of composite membranes, variations in the aspect ratio of nanocellulose derived from various sources and technologies are particularly appealing. Mechanical reinforcement is enhanced by a high aspect ratio paired with a high rigidity. Because CNCs are more rigid and CNFs have a much higher aspect ratio, a balance between these two key parameters is critical when selecting either CNCs or CNFs as reinforcing materials. While a high aspect ratio is one of nanocellulose’s most desirable characteristics, some applications require nanocellulose with uniform crystallinity and a lower aspect ratio. Post-treatment strategies such as ultrasonication, centrifugation, and filtration can be used to reduce heterogeneity in nanocellulose production [22].

The pore size of the randomly aligned CNF network, which allows the fabrication of membranes capable of rejecting pollutants of various sizes, is influenced by the diameter of the fibrils [23]. CNFs with diameters of 3–6 nm can provide a thin membrane barrier layer with a mean pore size on the order of 20 nm that is suited for MF and UF applications [24]. The mean pore size is linearly associated with the fiber diameter for an optimal barrier layer of polymeric nanofibers. As for CNF membranes, the hydration effect and solvent flexibilization may affect this relationship. Nonetheless, the link between pore size and fiber diameter can be beneficial in guiding CNF assembly in a nonwoven format.

### 2.2. Outstanding Mechanical Properties

One of the most promising features of nanocellulose is its superior mechanical properties [25]. The reported Young’s modulus, *E* of nanocellulose with a density, *ρ* = 1.60 g cm^−3^ might reach 220 GPa [26], which is significantly higher than glass fiber (E = 72.4 GPa, *ρ* = 2.60 g cm^−3^) [27] and comparable to Kevlar (E = 179 GPa, *ρ* = 1.47 g cm^−3^) [27] (Table 1). Nanocellulose is potentially stronger than stainless steel [28] with a specific Young’s modulus ranging from 65 to 85 J g^−1^, about three times higher than that of the steel (25 J g^−1^). Nanocellulose’s outstanding mechanical properties have inspired its use as a reinforcing material in the production of composite membranes. For environmental applications, especially water filtration, membranes with good mechanical properties are highly desirable to withstand the pressure exerted by flowing water [29]. As a result, numerous studies on the mechanical properties of nanocellulose reinforced composite membranes have been conducted in recent years as summarized in Table 2.

The reinforcing capabilities of nanocellulose in the matrix are determined primarily by nanocellulose composition and nanocellulose–matrix interactions. Hinestroza et al. (2020) [29] found that adding cellulose nanofiber (CNF) at a 50:50 ratio increased the elastic modulus and tensile strength of polycaprolactone (PCL) membranes significantly compared to PCL:CNF ratios of 60:40 and 80:20 and pure PCL. They postulated that the enhanced mechanical strength of the membrane with high CNF composition could be due to the effective PCL/CNF interaction. Similar reinforcing effects of CNF were observed by Liu et al. (2019) [31], in which the elastic modulus, tensile strength, and strain of graphene oxide were enhanced with the addition of 1–4% of CNF. Eom et al. (2020) [32] also demonstrated that increasing CNF content from 5% to 15% increased the elastic modulus and tensile strength of silk fiber. However, the strain was greatly reduced from 18.6% to 11.4%.

In several cases, the mechanical properties of nanocellulose-based membranes do not scale linearly with the composition of nanocellulose in the matrix [22]. Significant enhancement of mechanical properties can be achieved even at low nanocellulose concentrations (typically in the range of 1 to 10 wt%). The reduction in mechanical strength at higher nanocellulose concentrations is attributed to the nanocellulose aggregation in the matrix. For example, Ram et al. (2020) [33] found that the addition of 3 wt% polydopamine-coated nanocellulose to Nafion membrane resulted in a 32% increase in tensile strength from 11.5 to 15.15 MPa. A further increase in nanocellulose concentration to 7.5 wt% led to a slight reduction in the tensile strength to 13 MPa.

In the study conducted by Luo et al. (2020) [34], the tensile strength of lignin/hemicellulose/CNF composite increased with increasing CNF concentration from 10–50%, however, reduction in Young’s modulus was observed when the CNF composition in the matrix exceeded 20%. According to Patel et al. (2019) [3], Young’s modulus is mainly correlated with the nature of the nanocellulose such as the aspect ratio whereas the tensile strength is strongly dependent on the matrix behavior. The mechanical properties of nanocellulose-based membranes may also be affected by the type of nanocellulose used. Jiang et al. (2019) [35] revealed that spinifex CNC (s-CNC) and cotton CNC (c-CNC) have greater reinforcing capabilities on PAN nanocomposites than the spinifex CNF (s-CNF) at 0.1 wt% nanocellulose composition.

### 2.3. High Degree of Crystallinity

**Figure 3:**
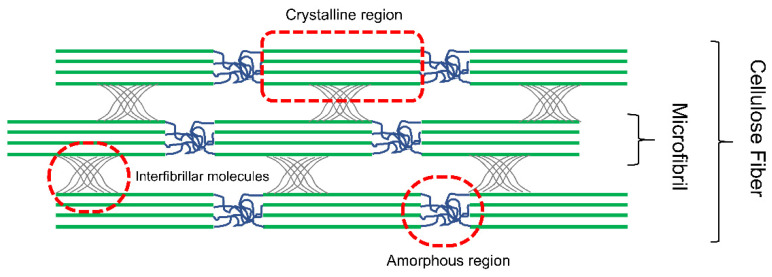
Crystalline and amorphous regions of cellulose as well as the interfibrillar network among cellulose molecules.

Nanocellulose is regarded as a high-performance biobased material with a high strength, stiffness, and thermal expansion. These exceptional features are attributed to the uniaxial molecular orientation of cellulose molecules in the microfibrils and their high crystallinity [40]. The crystallinity index (CI) of a material is defined as the mass ratio of the crystalline substance in the total dry sample on the basis of the crystallographic two-phase model [41,42,43]. CNCs which are mainly produced through acid hydrolysis generally have higher crystallinity than the CNFs due to the removal of most of the amorphous cellulose fraction. Figure 3 depicts the amorphous and crystalline domains of nanocellulose in the cellulose fiber. Depending on the amorphous content in the feedstock, production process, and processing conditions, the degree of crystallinity for CNCs varies between 54 to 88% [8,21,44,45]. CNCs made from H_2_SO_4_ treatment have lower crystalline values compared to those made from HCl. Apart from that, increasing the hydrolysis time enhances crystallinity as a result of the removal of amorphous regions. Crystallinity is also greatly influenced by the source of lignocellulosic biomass. Kunaver et al. (2016) [43] reported that the crystallinity of nanocellulose produced from cotton liner using the glycolysis method is 80%; whereas spruce wood, Chinese silver grass, and Eucalyptus wood nanocelluloses had a lower crystallinity (62.8 to 66%).

**Figure 4:**
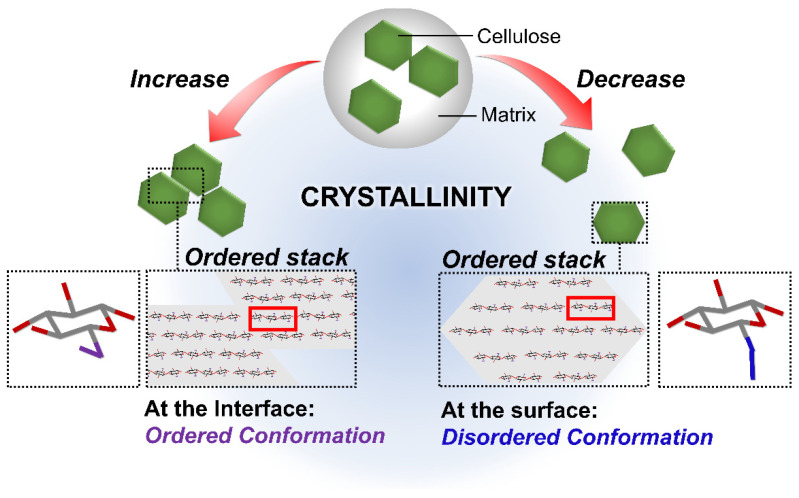
Mechanism of dispersion-induced disordering of the interfacial molecules between bundled microfibrils in a wood cellulosic structure that affect the crystallinity. Adapted from [47].

The interface between the bundled cellulose fibrils controls the crystallinity of CNFs, according to Daicho et al. (2018) [46]. The ordered arrangement of cellulose molecules at the interface or at the surface of the individual fibrils is achieved when the fibrils are densely bundled upon removal of the non-cellulosic components. When the bundled fibrils disperse upon disintegration to CNFs, the interfacial molecules become disordered (Figure 4).

For the development of composite membranes, the degree of crystallinity of the composite affects the accessibility of the OH groups on the surface of the membranes. Hinestroza et al. (2020) [29] developed a composite membrane consisting of CNF isolated from agave bagasse and polycaprolactone (PCL) nanofibers for water filtration. XRD was used to determine the membranes’ crystalline planes. When compared to the CNF (68.5%), the composite membrane had a slightly higher crystallinity (71.7%).

### 2.4. Tuneable Surface Chemistry

Nanocellulose has a reactive surface coated with an abundance of active hydroxyl groups, which allows for unprecedented opportunities for modifications to fit the desired applications. From a structural perspective, the reactive surface of nanocellulose is mediated by the three hydroxyl groups in each cellulose monomer. The hydroxyl group at the sixth position acts as a primary alcohol which is 10 times more reactive than the other hydroxyl groups. The hydroxyl group at the second position is found to be double to that of the third position, of which both serve as secondary alcohols [44,47]. This phenomenon is caused by the steric hindrance of each hydroxyl group, as the hydroxyl group in the sixth position is attached to a carbon atom that is only bonded to one alkyl group, whereas the carbon atom carrying the hydroxyl groups in the second and third positions is bonded to two alkyl groups [19,48].

Numerous scientific articles have been published focusing on strategies for tailoring nanocellulose’s surface chemistry or tuning its hydrophilic–hydrophobic characteristics [17,49]. The abundance of hydroxyl groups also induces high hydrophilicity of nanocellulose which has been proven to minimize membrane fouling by hydrophobic foulants [50]. However, due to its poor dispersibility in non-polar organic solvents and polymer matrices, pristine nanocellulose is typically limited to applications using hydrophilic or polar media [48]. As nanocellulose is often utilized as a reinforcing material in polymers, good dispersion of nanocellulose in the polymer matrices could be achieved through surface modification. CNF membranes’ surface chemistry can be tailored to enable pollutant removal by electrostatic interaction, size exclusion, or a combination thereof [31]. Surface charges and surface roughness of CNF membranes coated with a functional layer tend to improve for superhydrophilicity.

The goal of surface modification of a nanomaterial is to introduce new functional groups or important biological components to the nanostructure. Therefore, it is of utmost importance to highlight that the modification should be conducted in mild conditions in order to retain the other beneficial properties of pristine nanocellulose. Improvements in the material’s substitution degree and/or grafting efficiency should have no adverse effect on its structure, morphology, or crystalline characteristics [48]. Section 3.2 delves more into the mechanisms of nanocellulose surface modification approaches.

### 2.5. Anti-Fouling Properties

**Figure 5:**
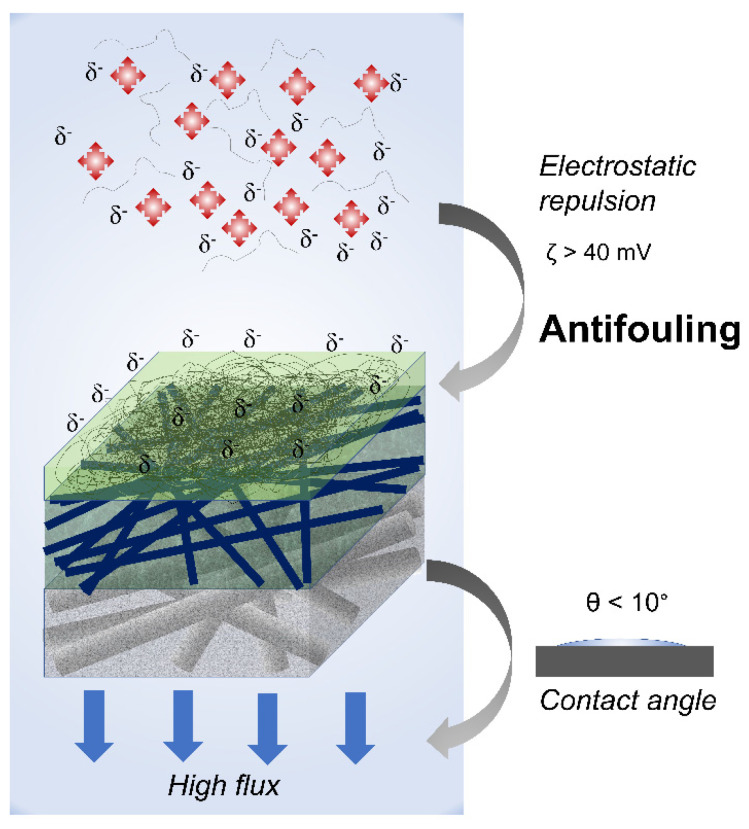
Illustration of antifouling behavior of nanocellulose barrier layer on nanocomposite membrane.

Fouling is one of the most serious issues in membrane applications. According to recent findings, nanocellulose’s high hydrophilicity could help to minimize membrane fouling. Liang et al. (2020) [51] used a solution-coating technique to create a PAN/PET composite ultrafiltration (UF) membrane with nanocellulose-integrated PAN as the barrier layer. The membrane’s anti-fouling capabilities were improved as a result of increased hydrophilicity, as measured by water contact angle measurements. The antifouling behavior of nanocellulose which acts as a composite membrane barrier layer is illustrated in Figure 5 [50]. Moeinzadeh et al. (2019) [52] observed a similar effect when nanocellulose was added into a PSF membrane. The addition of nanocellulose enhanced the membrane’s overall porosity and hydrophilicity. From the UF performance study, the PSF membrane with 1 wt% nanocellulose had a 43% higher water flux than the pristine membrane, high oil rejection (>98.2%), and an outstanding water flux recovery rate of 98% after filtering 250 ppm oil-in-water emulsion solution. The presence of hydrophilic hydroxyl on the membrane surface caused by the addition of NCs makes the membrane less susceptible to fouling during the treatment of oil-in-water emulsions.

Nanocellulose produced via a TEMPO-mediated method had better anti-fouling properties. Yang et al. (2021) [49] reported that the TEMPO-CNF-coated PAN membrane exhibited a high permeation flux (15–61 L m^−2^ h^−1^ at 0.5 psi) and rejection ratio (>98%), as well as good antifouling against the model pollutant (BSA) using simple hydraulic flushing. Good filtration performance of the TEMPO-CNF can be explained by the strong electrostatic repulsion between the negatively charged TEMPO-CNF. The findings imply the promising direction of using charged CNF as a barrier layer for antifouling membranes in wastewater treatment.

## 3. Recent Development in Nanocellulose-Based Membranes

The key aspects in the development of membranes based on nanocellulose are the surface modification strategies and preparation methods. The developed membrane should have optimal access to functional sites, combined with a high flux and high mechanical stability. In this section, various routes of nanocellulose functionalization and recent developments in nanocellulose-based membrane preparation techniques will be discussed.

### 3.1. Surface Modification Strategies of Nanocellulose for Membrane Development

**Figure 6:**
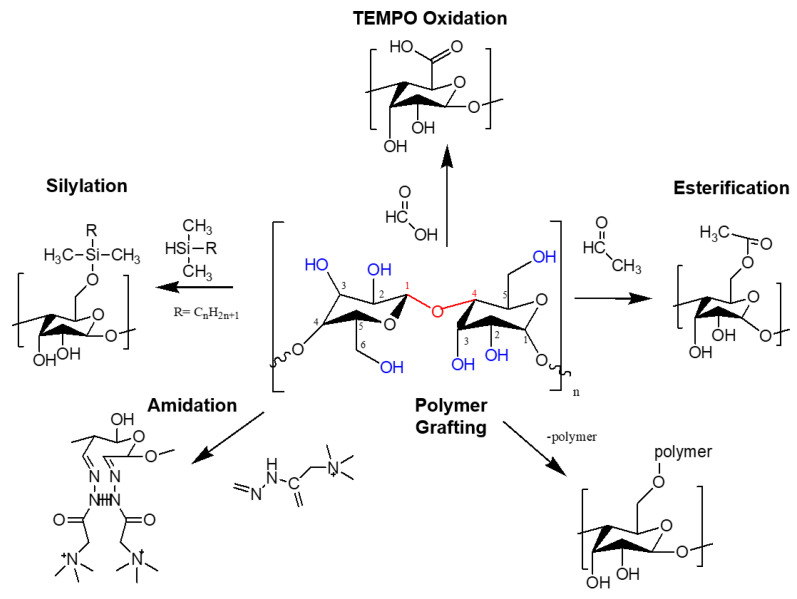
Chemical modification of nanocellulose.

Cellulose contains three hydroxyl groups with an anhydroglucose unit (AGU) in each monomer unit. Several chemical surface modifications can be developed on these hydroxyls group to improve its properties and selectivity that is relevant to its function. As cellulose has highly polar surfaces due to hydroxyls, their adsorptive compatibility with non-polar systems is poor and only limited to polar solvents and resins. Therefore, some strategies for the modification of nanocellulose are needed to control its surface chemistry without significantly altering the bulk structure of the nanocomposite membrane by minimizing the fibril entanglement and increasing their dispersion in the matrix. Chemical modification can take place in both homogenous and heterogenous states, although most chemical reactions in the heterogeneous state can only take place at the surface layer due to its limited dispersity. Oxidation, esterification, silane coupling, amidation, and polymer grafting are examples of common chemical modifications of nanocellulose (Figure 6) [53].

#### 3.1.1. Chemical Modification via Oxidation

**Figure 7:**
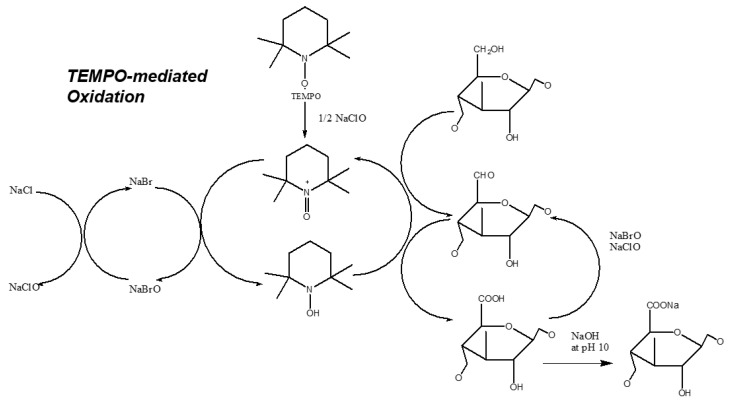
Mechanism of TEMPO-mediated oxidation.

The modification process that is commonly used is nitroxyl-based oxidation which involves 2,2,6,6-Tetramethylpiperidine-1-oxyl (TEMPO)-mediated oxidation, that is performed on nanocellulose to convert the hydroxyl groups into their carboxylic forms. Nitroxyl-based oxidation uses a stable nitroxyl radical TEMPO in the presence of NaBr and NaOCl. The C_6_ primary hydroxyls of cellulose are converted to C_6_ sodium carboxylate groups by dissolving catalytic amounts of TEMPO, NaBr, and nanocellulose in a polysaccharide solution at pH 10–11. The oxidation process begins when an NaClO solution is added as a primary oxidant (Figure 7). The recent development of nanocellulose modification via TEMPO-mediated oxidation uses ultrasonic treatment which causes the amorphous parts of cellulose to be removed selectively while the crystalline regions of cellulose macromolecules are preserved, compared to the conventional method which uses mechanical treatment to disintegrate cellulose fibers into microfibrillar [54]. This method produces nanocellulose films with a higher density and tensile strength [49]. Furthermore, there is also periodate-based oxidation, which is a reaction that used aldehyde to split bonds between vicinal carbons. This reaction uses sodium periodate (NaIO_4_) and potassium periodate (KIO_4_) as oxidants to oxidize secondary hydroxyl groups, breaking the chemical bond between C_2_ and C_3_, subsequently forming two aldehyde groups [18].

#### 3.1.2. Chemical Modification via Esterification

Ester-based surface-modified nanocellulose is formed by a series of condensation reactions between hydroxyl groups on nanocellulose and ester functional groups (-COO). Esterification is a two-step process involving the introduction of ester functional groups (-COO) to the surface of nanocellulose via homogeneous or heterogeneous processes. The heterogeneous reaction produces insoluble-modified nanocellulose that surrounds the crystalline core of unreacted cellulose chains in the media. Unlike in homogeneous conditions, the partially modified nanoparticles instantly split and dissolve in the media. Additionally, acetylation uses the esterification method which introduces an acetyl group (CH_3_-C (=O) to the surface of the nanocellulose. This process is dependent on the hydrophilic nature of the hydroxyl group on nanocellulose in amorphous and crystalline regions within the cellulose polymer chain, as it eventually turns to hydrophobic. Acetylation allows for optimal cellulose fiber dispersion in the polymer matrix during compounding, economical, convenient, rapid production times, and environmentally friendly [55].

#### 3.1.3. Chemical Modification via Silyation

**Figure 8:**
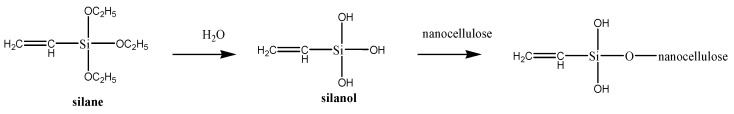
Silylation process of nanocellulose.

Silylation, or the silane-coupling reaction, is frequently used in CNCs to improve the interfacial adhesion of the polymer matrix and fillers in the composites. Furthermore, silylation could enhance the dispersion of nanocellulose, resulting in a more efficient transfer of mechanical and physical properties from the nanofiller to polymer [56]. In this process, silanes are used as coupling agents to stabilize the composite material, thereby reducing the amount of cellulose hydroxyl groups in the polymer matrix. First, hydrolyzable silyl groups react with moisture to form silanols. An acid-hydrolysis reaction then occurs in the silylation process, in which the interaction between the alkoxy group in silane and the hydroxyl group in nanocellulose forms a covalent bond via cross-linking between the polymer matrix and the nanocellulose, which later produces alcohol as a by-product (Figure 8). Condensation of Si-OH with C-OH results in permanent surface grafting after a temperature curing process.

#### 3.1.4. Chemical Modification via Amidation

Amidation is a process that is applied on the intermediate stage and targets mostly carboxylic groups of pre-oxidized nanocellulose for membrane modification. The amino group on the carbodiimide group such as *N*-Ethyl-*N*-(3-dimethylaminopropyl) carbodiimide hydrochloride (EDAC), polyethylenimine (PEI), and glycerol diglycidyl ether (GDE), functions as a positively charged domain that is easily protonated to form a strong electrostatic interaction with an anion pollutant. This amine-modified cellulose nanofiber is infused with a hydrophobic alkyl chain of TEMPO-oxidized cellulose nanofiber to enhance the hydrophobicity of the composite membranes. The final modified cellulose nanofiber has hydrophobic alkyls and isocyanate groups on the surface [57].

#### 3.1.5. Chemical Modification via Polymer Grafting

Polymer grafting involves two ways of modification; grafting to and grafting from routes. The ‘‘grafting-to’’ involves the attachment of cellulosic material linked to fully characterized and purified polymers via the coupling of prepared polymer chains, carrying reactive end groups onto pre-modified hydroxyl groups on the nanocellulose surface. On the other hand, the ‘‘grafting from’’ process requires an initiating agent to be mixed with a monomer and nanocellulose to induce polymerization that is performed directly on the surface of nanocellulose using hydroxyl groups as initiating sites [8].

### 3.2. Preparation Techniques of Nanocellulose-Based Membranes

**Figure 9:**
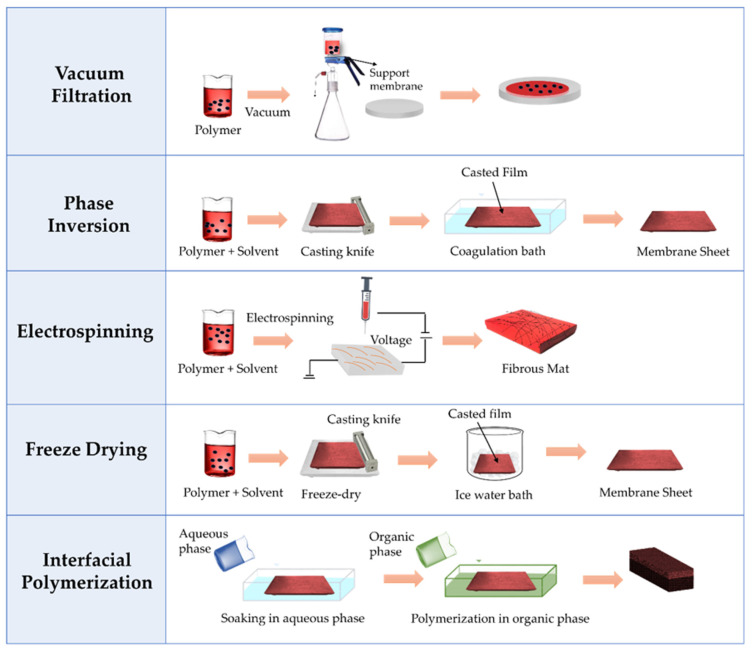
General procedures for the development of nanocellulose-based membranes through vacuum filtration, phase inversion, electrospinning, freeze drying, and interfacial polymerization.

There are various methods to fabricate nanocellulose-based membranes depending on their special properties and functions. Figure 9 illustrates the general procedures that are involved in the preparation of nanocellulose-based membranes [58].

#### 3.2.1. Phase Inversion

Phase inversion is an effective technique for fabricating asymmetric membranes [59]. This method uses a mechanism to transform a homogeneous solution to a solid-state material through thermal conversion, evaporation, or hydration. The pores’ morphology is directly influenced by the solvent exchange rate or precipitation rate, depending on the type of solvent being used. The same solvent is used during the synthesis of the nanocellulose and to obtain a mixture of polymer and nanocellulose to produce a casting solution. The final stage of the process involves pouring the casting solution onto the base membrane that will form a thin line followed by immersion in a coagulation bath. One method for preparing cellulose nanofiber membranes via phase inversion is to use an ionic liquid as the antifouling solvent [60]. The recent development of a novel nanocellulose composite membrane via a modified version of phase inversion technique using phosphotungstic acid and imidazole as a filler has found application in direct methanol fuel cells (DMFC) with improved properties [61].

#### 3.2.2. Vacuum Filtration

Vacuum filtration is the most straightforward and suitable technique for preparing layered structures of cellulose composite membranes, also referred to as the ‘nanopaper approach’. Composite membranes are frequently used in ultrafiltration applications. The composite membrane employs a thinner film layer for separation with a significant reduction in mass transfer resistance and enhanced membrane flux. In comparison to conventional commercial ultrafiltration membranes, direct vacuum filtration offers several advantages, including ultrahigh flux and low film resistance, making it suitable for a wide variety of industrial applications. Ultrafiltration membranes have been developed using a layer deposition technique aided by vacuum filtration, with filter paper (FP) serving as the support membrane and nanocellulose (NC) serving as the surface-barrier layer [62]. The technology can be utilized to synthesize low-cost NC/FP composite filtration membranes using a simple manufacturing procedure, resulting in high-performance materials. Nanopapers with a pore size of 19 nm were prepared using a vacuum filtration technique which utilized aluminum chloride (AlCl_3_) as a coagulating agent. This coagulant considerably enhanced their permeability, making them suitable for use in UF membranes [63,64,65,66]. Other than that, the preparation of novel nanocellulose composite membranes with the potential of treating wastewater is carried out by vacuum filtration in the wet state, which produces polydopamine/bacterial nanocellulose film membranes with high stability and fast water transport [66].

#### 3.2.3. Electrospinning

Electrospinning, as a well-established technology, is a route for creating membranes with an open yet continuously interconnected pore structure, a low basis weight, large effective surface area, and high effective porosity [65]. Electrospun nanocellulose exhibits a large number of isotropic pores evenly distributed among them due to its random arrangement. This characteristic makes it useful for a variety of applications [66]. A recent study on the production of cellulose acetate nanofibers via electrospinning on a wire mash demonstrated that the cellulose acetate fibers were selectively aligned vertically, horizontally, and then crossed at 45 degrees to the junction sites to create a surface template for future impregnation research. The bio-based membrane was then prepared by infusing the cellulose nanocrystal to the electrospun cellulose acetate fiber, resulting in improved mechanical properties, high water flux, hydrophilicity/anti-fouling and pollutant removal capability via adsorption, and size exclusion with a pore size in the microfiltration range [67]. Unlike the conventional method, the coaxial electrospinning method can also be carried out which uses multiple solution feed systems to electrospin two or more polymer solutions [68].

**Figure 10:**
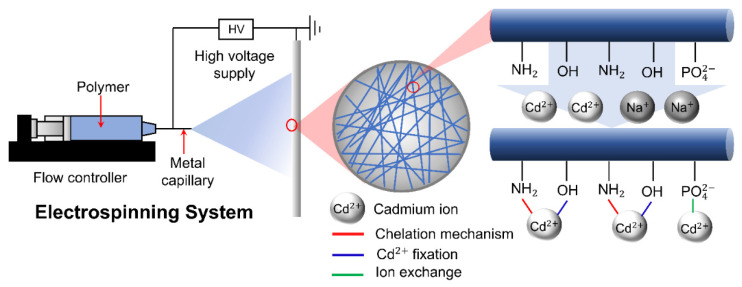
Setup of electrospinning system and mechanism of Cd^2+^ adsorption on the chitosan/PNC membrane.

The typical electrospinning system is comprised of a flow controller that regulates the flow rate of the fluid through a metal capillary connected to a high voltage supply. The resulting nanofiber is collected using a conductive substrate [69]. Brandes et al. (2019) [70] employed the electrospinning method to prepare a non-woven sorbent composed of chitosan and phosphorylated nanocellulose (PNC) for the removal of cadmium ions (Cd^2+^) from aqueous solution (Figure 10). The adsorption of Cd^2+^ occurred rapidly and reached equilibrium within 120 min which can be explained by the high affinity of amine and phosphate groups with Cd^2+^ at the surface of the electrospun membrane. It is therefore suggested that an electrospun nanofibrous membrane made from a nanocellulose composite is a promising material for the removal of heavy metal ions such as Cd^2+^.

#### 3.2.4. Interfacial Polymerization

Interfacial polymerization (IP) is a polymerization reaction between two monomers dissolved in immiscible solvents that occurs at the interface of a polymer substrate. Direct contact between monomers and rapid polymerization results in the formation of an ultra-thin layer film. The polymerization reaction will slow down once the monomers in two solutions separate which is controlled by the chemical kinetics of monomer diffusion through the film [71]. The process begins with soaking of the polymeric support for several minutes in the aqueous phase, followed by the introduction of the organic phase onto the membranes, where the reaction occurs. This method is applicable for the fabrication of reverse osmosis (RO) and nanofiltration (NF) membranes [53].

#### 3.2.5. Freeze Drying

The freeze-drying technique involves the use of a freeze dryer and casting of the polymeric solution on a flat casting surface to form a film. The casted film is then soaked in ice water to form a membrane sheet. A typical freeze-drying machine consists of a freeze-drying chamber with many shelves connected to heating units, a freezing coil coupled to a refrigerator compressor, and a vacuum pump [72]. This technique was used to prepare membranes for waste-water treatment using simultaneous citric acid as the cross-linking agent, but the results indicated that the membrane produced using freeze-drying has a lower mechanical stability compared to the conventional vacuum filtration method [73].

**Figure 11:**
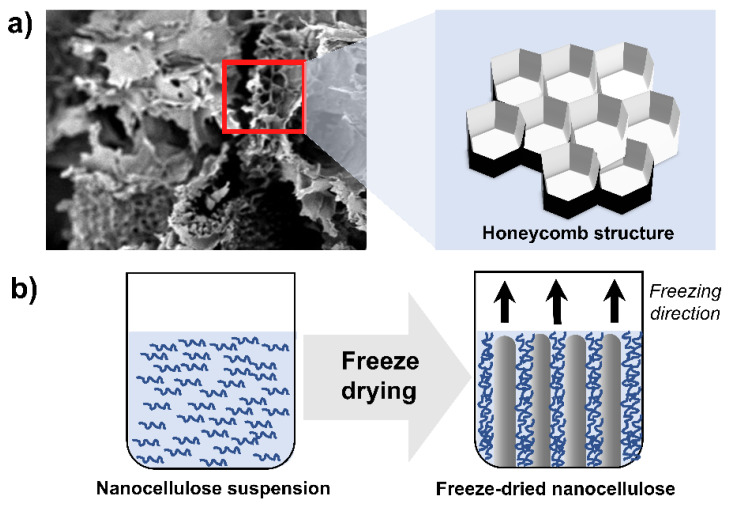
(**a**) Honeycomb structure produced by freeze drying of nanocellulose suspension and (**b**) mechanism of nanocellulose aggregation and ice-crystals growth along the freezing direction.

Previous studies have shown that NC tends to form bonds between ice crystals during the freeze-drying process [74,75]. The extent of bonding is highly dependent on the suspension concentration. The freeze-drying method was used as an ice-templating technique to create a membrane with a porous honeycomb-like structure (Figure 11). However, membranes produced using this method have relatively low surface area due to the aggregation of nanocellulose during the ice-templating process which consequently reduced the reinforcement effect of the nanocellulose material [76].

## 4. Environmental Applications of Nanocellulose-Based Membranes

### 4.1. Water Filtration

**Figure 12:**
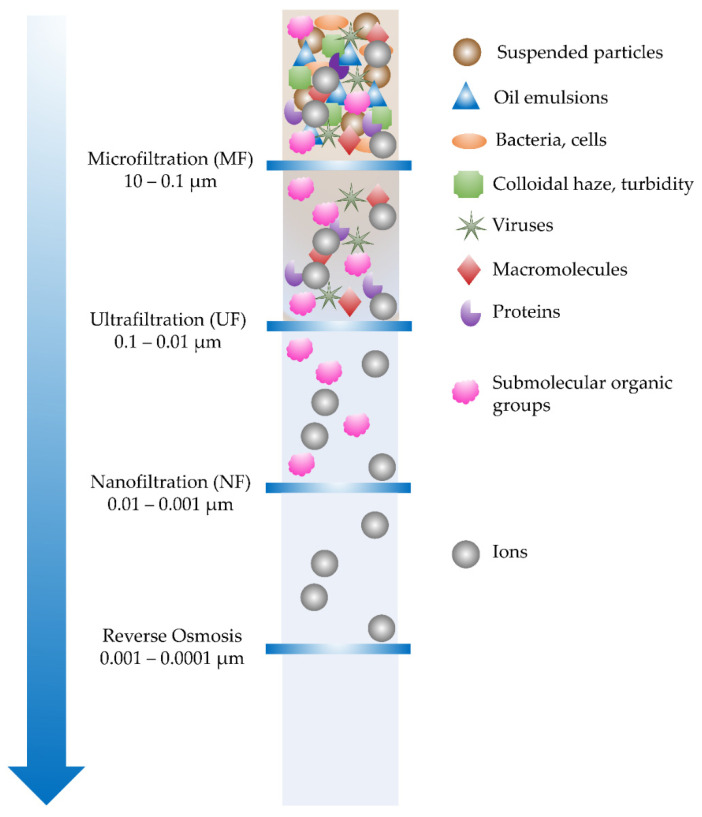
Type of membrane filtration: microfiltration (MF), ultrafiltration (UF), nanofiltration (NF), and reverse osmosis (RO).

Numerous technologies for water treatment have been developed, including physical, chemical, and biological treatments. Coagulation/flocculation, ion exchange, and chlorination/fluorination are all conventional methods that have a high operating cost and a low removal efficiency. These disadvantages can be overcome by pressure-driven membrane technologies that are capable of removing contaminants as small as 5 nm in size with a typical filtration efficiency of greater than 95% [73]. Figure 12 illustrates the four major types of pressure-driven membrane filtrations, including micro-, ultra-, and nanofiltration (MF, UF, NF), and reverse osmosis (RO) [18].

Recently, forward osmosis (FO) has been introduced to address the high energy consumption of pressure-driven membrane filtration systems. The FO membrane has a very small pore size of approximately 0.4–1.0 nm, which enables the removal of micro-pollutants and a high solute rejection rate. In FO, filtration occurs as a result of an osmotic pressure difference between the different sides of a semi-permeable membrane, which regulates the transport of fluid from a low concentration region to a high concentration region across the membrane. FO is particularly attractive because it does not require external pressure during the separation process and has a low fouling tendency [77].

A filter membrane with good selective permeability is the core component in membrane separation technology to separate the mixture solutes using the driving force derived from external energy or chemical potential difference [18]. Current material science breakthroughs provide a diverse range of raw materials for membrane development. Polymeric materials dominate the current membrane materials used in water and wastewater filtration. However, the primary disadvantages of most polymeric membranes are severe fouling caused by their hydrophobic nature, which has a detrimental effect on separation efficiency, flux, and membrane lifetime. Inorganic membranes, on the other hand, have a limited application due to their brittleness and high operating costs [73]. In this regard, nanocellulose materials are particularly promising for water filtration technologies due to their abundance, renewability, and biodegradability, as well as their exquisite properties of high water permeability, increased surface area, and superior mechanical properties. Table 3 summarizes different type of nanocellulose-based membranes for application in water filtration. 

The filtration efficiency of nanocellulose-based membranes is primarily governed by water permeation flux and the removal capacity of the specific pollutant. Membrane rejection is accomplished via two distinct mechanisms either through size exclusion or the membrane affinity to the target pollutant. The size exclusion process, as the name suggests, involves selective rejection of pollutants based on their size relative to the membrane pore size. The size-exclusion process is predominant in nanocellulose membranes, and the pore size is determined by the construction of a randomly aligned CNF or CNC network, which forms the “micro/nano-pores”. Affinity membranes, on the other hand, reject pollutants based on membrane–pollutant electrostatic interactions [63,78]. 

Varanasi et al. (2015) [79] fabricated biodegradable and recyclable ultrafiltration membranes using suspensions of CNFs, silica nanoparticles, and polyamide-amine-epichlorohydrin (PAE). The PAE adhered the negatively charged silica nanoparticles to the CNFs and improved the membrane’s wet strength. In addition, the nanocomposite membrane showed great potential for ultrafiltration with a water flux of 80 LMH and an MWCO of 200 kDa. Membranes fabricated via this process can be re-used in conventional paper recycling. Banana peel-derived bacterial nanocellulose has been used by Sijabat et al. (2019) [80] to develop a membrane for water filtration. 

Nanocellulose can be blended with metals, minerals, lignin, plasticizers, and even other cellulosic particles and work synergistically to improve the mechanical, rheological, and barrier properties of many polymeric systems [81,82]. Haafiz et al. [83] used the solution casting method to create poly(lactic acid) (PLA) bionanocomposites by incorporating the PLA into cellulose nanowhiskers (CNW) derived from oil palm-empty fruit bunches. The CNW/PLA nanocomposites demonstrated the ability to improve the properties of a polymer matrix at low filler loading and to enhance the tensile and thermal properties of bionanocomposites. Cellulose nanowhiskers have also been employed to coat alginate membranes for water purification. Mokhena et al. (2017) [83] fabricated a filtration membrane using two-layered nanoscale alginate nanofibres reinforced with maize cellulose nanowhiskers. The nanocellulose reinforcement produced a membrane with enhanced mechanical properties, and the membrane was able to completely remove water pollutants via size exclusion. The membrane was shown to reject 80% of chromium (Cr(VI)) at a pH of 11, indicating that it is suitable for short-term wastewater treatment and residential water purification.

To fabricate the support layer of thin-film composite membranes, a metalized nanocellulose composite was developed using silver and platinum nanoparticles as additive materials. The fabricated membranes demonstrated increased water flux and selectivity [84]. Chitosan and nanocellulose, two naturally occurring biodegradable polysaccharides, were used to create composite membranes for the removal of heavy metals (chromium ion) [85]. The nanocellulose was extracted from sugarcane bagasse via acid hydrolysis followed by a delignification procedure. The prepared membrane performed consistently well in removing chromium ions at concentrations ranging from 87 to 29 ppm over a four-cycle period. Nanocellulose/filter paper (NC/FP) composite filtration membranes were developed by Wang et al. (2019) [62] using a layer deposition method. The NC/FP composite membranes with a higher nanocellulose composition and aspect ratio exhibit improved rejection rates with reduced water flux.

**Table 3:** Different types of nanocellulose-based membranes for water filtration.

Materials	Method of Preparation	Filtration Process	Sample	Performance	Reference
Nanocellulose/filter paper (NC/FP) composite membrane	Vacuum filtration	UF	Oily wastewater	Up to 97.14% retention rate; 46,279 L m^−2^ h^−1^ flux	[62]
Metalized nanocellulose (silver and platinum as additive)	Vacuum filtration	FO	Nanopure water, urea, and wastewater	High water flux and solute rejection with wastewater sample	[84]
Cellulose acetate membrane	Phase separation	UF	Wastewater	207.32 L m^−2^ h^−1^ pure water permeability; 90.56% flux recovery ratio	[86]
Cellulose acetate/copper oxide nanoparticles	Wet precipitation	UF	Wastewater	Improved hydrophilicity, water permeation, BSA separation, and antifouling performance	[87]
Cellulose membrane	Thermally inducedphase separation	MF	Oily wastewater	99% rejections to peanut oil and pump oil nanoemulsion	[88]
Nanocellulose as modifer for hollow fiber	Addition of nanocellulose to internal coagulant	UF	Dye	Permeability increased 1.5 times; rejection increased from 96 to 99%	[89]
Biocellulose nanofibers membrane	Biosynthetic process followed by a purification step involving alkali treatment	NF	Emulsified oily wastewater	99% separation efficiency; permeate flux recovery ratio >94%	[90]
Carbon nanofiber (CNF)/cellulosic membranes	Carboxylic and amine functionalized CNFs	FO	Desalination	15 L m^−2^ h^−1^ water flux	[91]
Cellulose triacetate (CTA) and novel thin film composite	Calcium alginate as a model foulant.	FO	Desalination	Physical cleaning was more efficient	[92]
CNC and TOCNF coated polyethersulfone (PES) membrane	Layer-by-layer deposition	MF	Water	Improved antifouling and antibacterial properties	[93]

Apart from having efficient separation properties, nanocellulose also exhibits an antifouling behavior. The addition of suitable additives to cellulose changes its permeability and antifouling properties. Vetrivel et al. (2020) [87] customized cellulose acetate ultrafiltration membranes with copper oxide nanoparticles for efficient separation with antifouling behavior. The CuO nanoparticles were synthesized from cupric nitrate using a wet precipitation method. The cellulose acetate nanocomposite membrane with 0.5 wt% of hydrophilic CuO exhibited an enhanced PWF of 118.6 Lm^−2^ h^−1^ due to the improvement in porosity and water uptake. Its superiority for antifouling was demonstrated by a BSA separation rate of 95.5% and flux recovery ratio of 94.7. Kong et al. [94] discovered an ultrafiltration membrane prepared with improved antifouling properties. The ultrafiltration membranes were produced via the phase inversion technique using hydrophilic TEMPO-oxidized cellulose nanofibrils (TOCNFs) as modifying agents. The addition of TOCNFs improved the membrane’s permeability and mechanical properties. Aguilar-Sanchez et al. (2021) [93] reported that the coating of TOCNFs on a commercial polyethersulfone (PES) microfiltration membrane via the layer-by-layer method could enhance the membrane’s antifouling and antibacterial properties.

### 4.2. Environmental Remediation

Nanocellulose is of great interest in environmental remediation owing to its cost-effectiveness and renewable adsorption [95]. Extensive research has been conducted as evidenced by many reviews available related to environmental remediation by nanocellulose [96]. For instance, Tshikovhi et al., (2020) [95] reviewed the application of nanocellulose-based composites for the removal of various organic and inorganic contaminants from wastewater. The review discussed the building blocks, structure, properties, and isolation of nanocellulose-based composites with various types of reinforcements. Qiao et al., (2021) [14] addressed the surface modifications of nanocellulose-based adsorbents with various effective adsorption groups and polymer grafting as well as the fabrication of a hybrid composite. Recent progress in the synthesis, characterization, and possible applications of nanocellulose produced from various types of lignocellulosic biomass was reviewed by Marakana et al., (2021) [9]. Additionally, surface modification routes such as esterification, amidation, silylation, etherification, and carbamation to create advanced nanocellulose materials for desired applications were also discussed. Reshmy et al., (2022) [97] recently published a review on the prospect of nanocellulose as biosorbent, scaffold, and membrane for the bioremediation of heavy metals. They focused on methods to design nanocellulose biosorbents with the goal of improving the adsorption efficiency according to specific contaminants. In this section, recent research on the application of nanocellulose as an adsorbent for organic and inorganic pollutants, as a photocatalyst, and for gas separation will be reviewed.

#### 4.2.1. Nanocellulose as Adsorbent

Water pollution is becoming a severe problem due to aggressive industrialization. There are two categories of water pollutants namely, organic and inorganic. Organic water pollutants include dyes, pesticides, drugs, organic solvents, among others. Inorganic water pollutants, on the other hand, include metals, fertilizers, acids, alkalis, and salts. Organic dyes and heavy metal ions such as Pb(II), Cd(II), Cu(II), Fe(III), Cr(VI), Hg(II), and Co(II) are hazardous pollutants that cannot be biodegraded [98].

Nanocellulose has recently emerged as a promising adsorbent for the removal of water pollutants or contaminants as summarized in Table 4. Ngwabebhoh et al. (2019) [99] have demonstrated the successful application of amino-modified nanocellulose (A-NCC) for the removal of boron from aqueous media. A-NCC exhibits a strong affinity for boron with a maximum removal efficiency of 86.73% which was achieved at pH 7 within 120 min. Correspondingly, the nanocellulose adsorbent showed a significant capacity for reusability capacity for at least four successive adsorption/desorption cycles with minimum loss of recovery efficiency, indicating its great potential for boron removal in water remediation. Utilization of empty fruit bunch (EFB)-based nanocellulose (NC) prepared and functionalized with activated carbon for water remediation has been reported by Septevani et al., (2020) [100]. Adsorbent formulated with 2 wt% nanocellulose demonstrated a selective and notable metal adsorption capacity of 24.94 mg/g with 86% efficiency at a very short contact time of 3 min. They asserted that the value is almost two-fold higher than that of the rice-straw nanocellulose. The EFB/NC nanocomposite membrane could retain its adsorptive capacity after reuse.

Mutar and Jasim (2021) [105] reported the removal of disperse yellow (DY) dye from its aqueous solution by utilizing nanocellulose prepared from microcrytalline cellulose. According to the Giles classification, the adsorption isotherm of the (DY) dye was (S-type), which is well-fitted to the Freundlich equation. From the kinetic data, the adsorption process obeys the pseudo-second-order. In another application, Nematollahi et al. (2022) [106] investigated the use of cinnamon nanocellulose synthesized via acid hydrolysis as a catalyst for the decolorization of methyl orange as an azo dye. A decolorization efficiency of 40.64% was achieved which is two times higher than that of an uncatalyzed system at room temperature. The nanocellulose also possessed structural and morphological stability. Very recently, an interesting finding on the excellent adsorption capacity (14.71 mg/g) of a nanocellulose-based membrane at extremely acidic pH (pH 1.22) was reported by Wu et al. (2022) [110]. The membrane was prepared using a combination of chitosan and CNF via the gel-casting method. They postulated that the high stability of the membrane against a strong acidic environment is attributed to the ionic and hydrogen-bonding interactions between the chitosan and CNF.

#### 4.2.2. Nanocellulose as Photocatalyst

Organic dyes are commonly used in the textile and paper industries. The process generates a large volume of toxic waste which is difficult to degrade, posing a threat to the environment and human health. Various methods have been proven to be effective for treating the organic dyes in wastewater such as non-covalent adsorption, membrane filtration, ion exchange, and chemical oxidation. Unfortunately, these strategies may lead to insufficient pollutant degradation, resulting in secondary pollution [112]. Titanium dioxide (TiO_2_) has been widely used as a semiconductor photocatalyst because of its reactivity, stability, and cost effectiveness. However, in practical photocatalytic applications, aggregation of TiO_2_ nanoparticles is often inevitable, and they are also difficult to recycle. The use of nanocellulose as a supporting material has been suggested as a promising solution to overcome the aforementioned problem.

**Figure 13:**
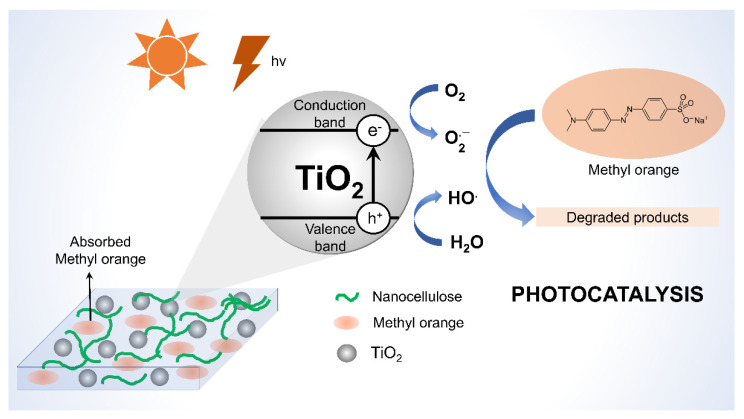
Mechanism of photocatalytic degradation of methylene blue by TiO_2_/GO/cellulose membrane.

The construction of a TiO_2_-based composite would be beneficial to achieve better photocatalytic performance [18]. Voisin et al., (2021) [113] reported the fabrication of CNC–TiO_2_ hybrids with an improvement in photocatalytic properties in suspension compared to the as-prepared control anatase TiO_2_ owing to a better dispersibility and colloidal stability. CNC’s interfacial properties were also preserved, enabling organic dye degradation in Pickering emulsions. Interestingly, the emulsions could be freeze-dried into versatile aerogels that degraded both aqueous and organic dyes efficiently. Liu et al. (2021) [114] employed a similar strategy to synthesize an anatase TiO_2_/nanocellulose composite for photocatalytic degradation of methyl orange in an aqueous solution. The composite exhibited good morphological characteristics and an anatase crystal structure, with the specific surface area being proportional to the amount of CNFs used. Within 30 min, a 99.72% degradation rate of methyl orange was achieved with no noticeable activity loss observed after five cycles. The findings could aid in the development of effective photocatalysts for the treatment of organic dye effluent. The proposed mechanism of photocatalytic degradation of methylene blue by the action of TiO_2_/nanocellulose is depicted in Figure 13.

Other researchers used a hydrolysis–precipitation approach to synthesize CeO_2_/TiO_2_ templates using nanocellulose. The photocatalytic performance of CeO_2_/TiO_2_-NCC was evaluated for the removal of Rhodamine B (RhB), methyl orange (MO), and the reduction of Cr (VI). Complete removal of a 250 mL and 50 ppm MO and RhB solution, as well as the reduction of a Cr(VI) solution, were accomplished in 70, 50, and 60 min, respectively [98]. Despite the widespread usage of TiO_2_ and other traditional photocatalysts such as zinc oxide (ZnO), they have been found to be ineffective under solar irradiation. This is due to the large bandgaps (3.2 eV) that allow only 4% of the potential 47% UV–Vis light radiation to be absorbed. Alternatively, the photocatalytic activities of silver phosphate (Ag_3_PO_4_) have been extensively studied for the removal of organic contaminants in the environment. Lebogang et al. (2019) [112] synthesized an Ag_3_PO_4_/nanocellulose composite using an in situ facile method for the photocatalytic degradation of methylene blue and methyl orange under solar irradiation. The photocatalytic degradation rate of MO was 90% in DI and 70% in wastewater over the same period. Owing to the good compatibility between the particles, Ag_3_PO_4_ and nanocellulose, the nanocomposite will be a good candidate for the development of photocatalytic membranes for water and wastewater treatments.

On other occasions, Nahi et al. (2020) [115] reported the green synthesis of a visible-light-driven photocatalyst made from a zinc oxide/nanocellulose composite (ZnO/NC) for the photodegradation of Enrofloxacin (EF), an antibiotic commonly used in veterinary medicine to treat animals with bacterial infections. The incorporation of nanocellulose enhanced the bandgap to the visible region, allowing degradation of the EF under visible light. Photo degradation was due to electron–hole interactions. The optimum pH was found to be 5, and the ZnO/NC dose was optimized to be 0.20 g/L. Equilibrium was attained at 120 min with a maximum degradation efficiency of 97%. Recent examples of the application of nanocellulose-based photocatalysts for pollutant degradation are shown in Table 5.

#### 4.2.3. Nanocellulose for Gas Separation

**Figure 14:**
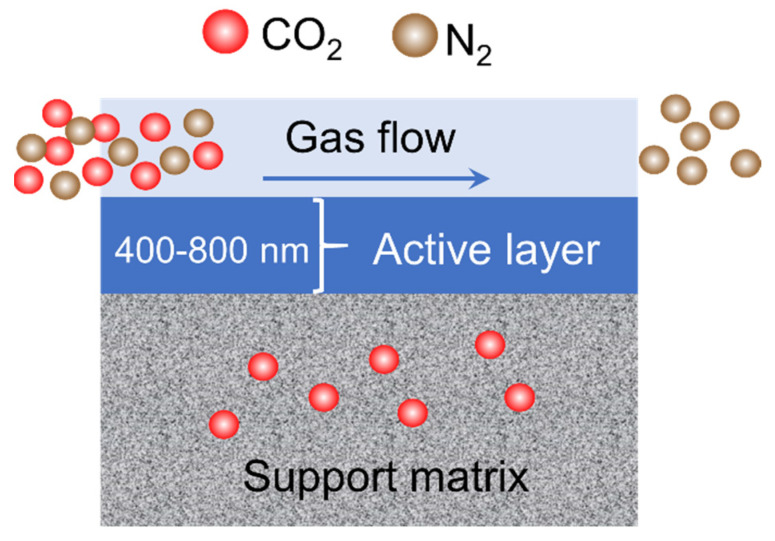
Mechanism of CO_2_/N_2_ separation using PVA/CNC as active layer.

Another important application of nanocellulose-based membranes is gas separation. CO_2_ is the most prevalent anthropogenic greenhouse gas [121]. Membranes selectively allow CO_2_ to pass through from the feed side to the permeate side. A polyvinyl alcohol (PVA)/crystalline nanocellulose (CNC) nanocomposite membrane CO_2_/CH_4_ separation was fabricated by Jahan et al. (2018). The addition of 1.5% CNC to the PVA remarkably improved the membrane’s selectivity and permeance. In the study conducted by Torstensen et al. (2019) [122], nanocellulose was employed as an additive to the PVA membrane for CO_2_/N_2_ mixed-gas separation. They discovered that the performance of the PVA/CNC membrane is comparable to that of PVA/carbon nanotubes, implying that CNCs can be used as an alternative to CNTs. Figure 14 depicts the mechanism of CO_2_/N_2_ separation employing PVA/CNC as the active layer. Helberg et al. (2021) [47] synthesized high-charge nanocellulose (H-P-CNF) and high-charge nanocellulose with screened size (H-P-CNF-S) and integrated them into a PVA matrix for CO_2_/N_2_ separation. The CO_2_ permeability of the H-P-CNF-S/PVA membrane was 160% higher than that of the neat PVA. Their findings suggest that the high charge and small size of nanocellulose are useful properties for CO_2_ separation under humid conditions.

### 4.3. Pollutant Sensors

These days, sensor technologies are gaining a lot of attention, thanks to the rapid expansion of the Internet of Things (IoT) and the Industrial Revolution 4.0. Sensors detect and transform physical, chemical, and biological changes in their environment and convert them into analytical signals. Nanocellulose, being a natural nanomaterial, is crucial in the development of new sensors, especially in the context of developing a multidimensional architecture [123,124,125,126]. Table 6 summarizes the recent developments of nanocellulose-based sensors including electrochemical, optical, colorimetric, fluorescent, and biosensors for the detection of various types of pollutants such as heavy metal ions, water-soluble gases, minerals, and salts.

Nanocellulose has a number of hydroxyl groups that can be exploited to accommodate binding sites for selective analyte species adsorption, which increases electrochemical sensor selectivity, sensitivity, and durability. Taheri et al. [124] discovered that an electrochemical sensor based on a D-penicillamine-anchored nano-cellulose (DPA-NC)-modified pencil graphite electrode exhibited good selectivity and sensitivity for copper ion in tap and river water samples, with a detection limit in the picomolar range (0.048 pM). The porous structure of the modifier and the formation of a compound between copper ions and nitrogen or oxygen-containing groups in DPA-NC may have contributed to the improved electrochemical responsiveness of the modified electrode. Shahi et al. [125] found that the as-prepared CNF and (TEMPO)-oxidized cellulose nanofibers/glycerol (TOCNF/G) sensors demonstrated good gas sensing capability against acetone, ammonia, methane, and hydrogen sulphide after surface modification with TEMPO-mediated oxidation and glycerol. The presence of numerous carboxyl and hydroxyl groups was responsible for the change in ionic conductivity of the sensors in response to gas exposure.

TOCNF has also been used for the development of colorimetric sensors for the detection of fluoride ions. Fluoride in the soil, water, or even the environment can harm plants and has major effects on crop development, growth, and maturity, thus calling for effective monitoring and detection. Cross-linking of the TOCNF with chemically modified branched polyethyleneimine (bPEI) yielded a heterogeneous colorimetric sensor that enabled naked-eye detection of fluoride ions with concentrations of up to 0.05 M in DMSO. Moreover, it is highly selective towards fluoride ions over chloride, phosphate, and acetate ions [101].

Pouzesh et al. developed an optical plasmonic chemosensor for detecting cyanide in environmental and industrial water samples. Cyanide ion (CN^-^) is one of the most hazardous ions for humans and microorganisms, even at low concentrations. The sensor was prepared by embedding stable copper nanoparticles in the nanocellulose framework (ECNPs-NC) by a direct chemical reduction of copper ions onto the nanocellulose using small amounts of sodium borohydride (NaBH4). Changes in the surface plasmon resonance absorption intensity of the ECNPs-NC film were monitored by a UV–Vis spectrophotometer. The linear range of absorption intensity was 0.25–0.40 μg mL^−1^ with a detection limit of 0.015 μg mL^−1^. The significant advantages of this method are its simplicity, no organic synthesis and solvent required, and sensing in pure aqueous media.

Besides chemical, electrochemical, and optical sensors, biosensors also have a vital role in detecting pollutants. CySense, a biosensor based on a red-fluorescent-sensing protein, known as cyanobacterial C-phycocyanin (CPC), and TOCNF has been fabricated by Weishaupt et al. [127] for detection of copper ions. The covalent binding of CPC to TONFC stabilized the sensing molecule without the need for preservatives. The film production method used is reproducible in which the morphological and optical properties can be analyzed using techniques such as fluorescence spectrometry or microarray laser scanning. The ability to laser cut such CNF-based composite films could further aid their use in a variety of sensing applications.

The discovery of nanopaper was another significant step forward in the development of pollutant sensors based on nanocellulose. Nanopaper is a type of thin membrane constructed of cellulose nanofiber that opens up new avenues for producing simple, quick, low-cost, and single-use analytical instruments for a variety of applications, including environmental monitoring [128]. The detection of iodide in seawater using nanopaper made from bacterial CNF is a recent example. Photoluminescence properties are obtained by embedding carbon-quantum dots in nanopaper. The nanopaper can be placed in a microcuvette for spectrofluorometer iodide sensing. Another application of nanopaper based on bacterial CNF is for the enantioselective detection of chiral compounds. In situ-synthesized silver nanoparticles were embedded in nanopaper, which displayed a significant color change from yellow to purple-brown in both the aqueous phase and the nanopaper upon aggregation of the silver nanoparticles induced by the chiral compounds [133,134]. Nanopapers can also be used to host nanoparticles activated by surface-enhanced Raman spectroscopy (SERS) such as silver and gold, for analyzing organic pollutants and aquatic pathogens [135,136,137].

### 4.4. Energy Devices

The world is currently being confronted with a number of environmental issues such as global warming, resource scarcity, and severe environmental pollution. Therefore, the development of energy devices that are both efficient and environmentally benign is crucial [137,138]. In this context, nanocellulose-based materials may hold great promise in the development of energy devices such as fuel cells [139,140,141,142], solar cells [140,141,142,143], and generators [144,145,146,147] to address the core challenges related to environmental sustainability.

#### 4.4.1. Fuel Cells

**Figure 15:**
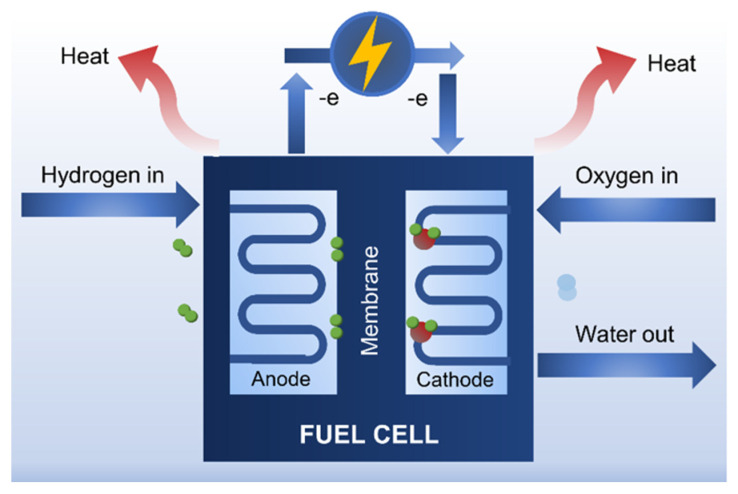
Mechanism of electricity generation in fuel cell.

Fuel cells are electrochemical energy conversion devices that generate electricity from the chemical energy of fuels (such as hydrogen) (Figure 15). It has the potential to be one of the most promising “green” power sources for road and stationary applications [148,149,150,151]. Nafion is used extensively as a polymer electrolyte membrane (PEM) for fuel cells due to its outstanding thermal and mechanical properties, as well as good proton conductivity. The PEM is an important component of the fuel cell that allows safe and selective proton transport from the anode to the cathode [33]. Nafion, however, has some disadvantages such as high production costs, the formation of toxic intermediates, and most importantly, proton conductivity loss when operating at high temperatures (>100 °C). Therefore, nanocellulose-based membranes in the form of thin film or sheets have emerged as promising low-cost alternatives to Nafion, with additional benefits such as biodegradability, good gas barrier and mechanical properties, and acidic oxygen functional groups [141,142,152,153].

Rogalsky et al. (2018) [139] fabricated a fuel cell for high-temperature application using PEM based on a BNC membrane with protic ionic liquid N-butylguanidiniumtetrafluoroborate (BG-BF4). A significant enhancement of ionic conductivity from 4.5 × 10^−4^ Scm^−1^ to 5.2 × 10^−2^ Scm^−1^ at 180 °C was observed when the concentration of BG-BF4 was increased from 80 wt% to 95 wt%. However, the tensile strength was reduced from 35 to 6 MPa. To evade this undesirable effect, aniline was oxidatively polymerized into the BNC matrix, resulting in considerably high ionic conductivity (4 × 10^−3^ Scm^−1^ at 180 °C) with exceptional stability.

To serve as a proton exchange membrane in fuel cells, the nanocellulose surface chemistry can be tuned to enhance its proton conductivity [142,153]. The incorporation of sulfonic acid-functionalized CNCs to sulfonated poly(aryl ether ketone)s with carboxylic acid groups (SPAEK-COOH-x) resulted in a slight improvement in the proton conductivity of the membrane [153]. This ‘performance-enhancer’ effect of the functionalized nanocellulose was more pronounced in sulfonated fluorenyl-containing poly(ether ether ketone ketone)s (SFPEEKKs), with a 61.2% improvement in proton conductivity at 90 °C compared to that of the pristine membrane [144]. This was due to the synergistic interaction between the hydrogen bond networks and proton conduction paths provided by the SO_3_H/–OH groups available at the CNCs surface and SO_3_H groups at the membrane matrix backbones [153]. CNCs-based membrane functionalized with sulfonic acid and 1,2,4-triazole exhibited high proton conductivity of 13 mScm^−1^ at 120 °C in anhydrous condition [154,155].

#### 4.4.2. Solar Cells

Clean solar energy can be converted into electricity or heat with the aid of solar cells. However, the high material costs of photovoltaics limit the application of solar cells. Because the substrate contributes to 25–60% of the overall material cost, adopting low-cost substrates is a viable solution to solve this problem [109,117]. Nanocellulose-based material is a revolutionary green substrate for solar cells owing to its prospective low cost and tunable physical and chemical properties. A number of studies have focused on improving the performance of solar cells by utilizing nanocellulose-based substrates and optimizing the cell fabrication process [144,146,156,157,158,159].

Gao et al. [158] fabricated biodegradable perovskite solar cells (PSCs) with a high-power conversion efficiency by using acrylic-coated transparent nanocellulose paper. The PSCs exhibited a power conversion efficiency of 4.25%, high power per weight ratio (0.56 W g^−1^), and exceptional stability (more than 80% original efficiency retained after 50 times bending test. Voggu et al. [146] developed a nanocrystal photovoltaic device (PV) using paper made from *Gluconacetobacter hansenii* CNF as the substrate and Copper Indium Selenium (CuInSe_2_) nanocrystals as the absorber. The PV demonstrated good thermal and air stability, with high power conversion efficiencies of up to 2.25%. Furthermore, the PV had high mechanical flexibility without degradation. Moreover, the device could retain its performance even after 100 cycles repeated bending test to a radius as small as 5 mm.

#### 4.4.3. Nanogenerators

**Figure 16:**
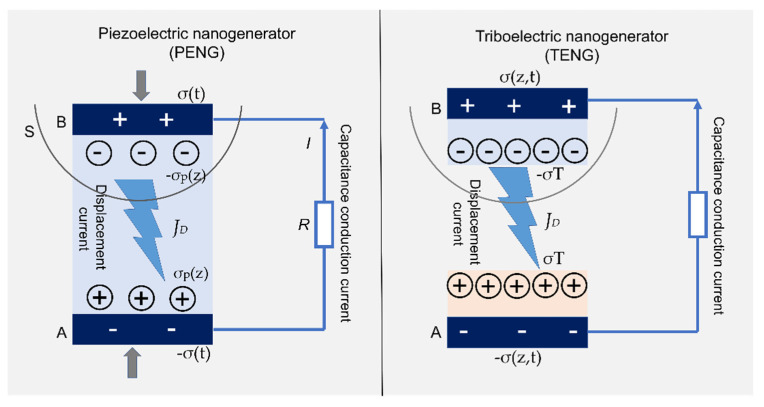
Schematic representation of piezoelectric nanogenerator (PENG) and triboelectric nanogenerator.

Nanogenerators can transform mechanical energy into electricity, making them an excellent alternative power source for portable electronics and unattended devices. Based on the principles of electricity generation, nanogenerators can be classified into triboelectric nanogenerators (TENGs) and piezoelectric nanogenerators (PENGs) (Figure 16) [33,149,160]. Yao et al. [148] were the first to develop flexible and transparent TENG by combining a nanocellulose-based film that served as a triboelectric material with fluorinated ethylene propylene (FEP) as a negative material. The film exhibited a high surface roughness (~300 nm), which provided a large surface area for interaction and generation of electrostatic charges. The TENG demonstrated comparable performance (0.56 mW at 1 MΩ) to that of synthetic polymer-based TENGs. Another transparent, flexible, and biocompatible TENG was fabricated by Kim et al. [150] consisting of bacterial nanocellulose and Cu foil. The TENG showed an accumulative charge and peak power density of ~8.1 μC/m^2^ and ~4.8 mW/m^2^, respectively, at 1 MΩ load resistance.

The tribopolarities of nanocellulose have to be tailored to generate environmentally friendly all-nanocellulose TENGs. Thanks to the abundant hydroxyl groups on the nanocellulose, its surface chemistry can be easily tuned using simple wet chemistry. Nitro and methyl groups have been introduced individually into the CNF to modulate the tribopolarities without altering their physical characteristics [149]. The nitro-CNF has a negative surface charge density of 85.8 μC/m^2^. As for the methyl-CNF, it has a positive surface charge density of 62.5 μC/m^2^. Pairing these two films resulted in a TENG with an average voltage and current value of 8 V and 9 μA, respectively, comparable with TENGs made using florinated ethylene propylene.

Piezoelectric composite paper made from ferroelectric BaTiO_3_ nanoparticles and bacterial cellulose showed an excellent piezoelectric performance [147] with a peak voltage, peak current density, and maximum power density of 14 V, 190 nA/cm^2^, and 0.64 μW/cm^2^, respectively. Nanocellulose-based PENGs, on the other hand, have a substantially lower output than nanocellulose-based TENGs.

## 5. Challenges and Opportunities

### Challenges in Nanocellulose Production and Application

From their structural perspective and outstanding properties, nanocellulose-based membranes are indeed very promising for a wide range of environmental applications. However, these are the limitations and challenges in the utilization of nanocellulose that need to be addressed:

i. Cost-Effective Production and Upscaling

The structure and properties of nanocellulose in relation to membrane performance can be controlled using the methods presented in this review. Nonetheless, finding cost-effective industrial processes for nanocellulose synthesis and upscaling is critical for commercialization. Several companies such as Diacel FineChem Ltd., Tokyo, Japan, and Celluforce, Quebec, Canada, are now focusing their efforts on cost-effective and large-scale production of nanocellulose which is expected to provide the market with sufficient amounts of nanocelluloses [21,161].

ii. Complex Preparation Process

The production of nanocellulose from various lignocellulosic biomass mainly relies on the multi-step process involving pre-treatment with sodium hydroxide, bleaching with a chlorine-based reagent, and sulfuric acid hydrolysis at high concentration (~65%). This is due to the recalcitrant nature of lignin and hemicellulose embedded in the cellulose framework. While this method has been employed for decades, its environmental impact is still a major concern. Environmentally friendly manufacturing processes as well as disposal routes for nanocellulose are deemed necessary for the commercial implementation of nanocellulose, particularly in membrane technology [22].

iii. Dispersion of Nanocellulose

Though stable nanocellulose dispersion in aqueous solutions can be achieved by introducing negatively charged sulfate groups via sulfuric acid hydrolysis, their separation from the water system is challenging and necessitates the addition of salt or pH alteration to recover them after the water treatment process. Moreover, the dispersion of nanocellulose in hydrophobic polymer matrices (membranes) remains a critical issue. An important strategy to combat this issue is by surface grafting of nanocelluloses with low-molecular-weight polymers [162].

iv. High Tendency of Clustering

A common challenge associated with the preparation of membranes based on nanocellulose is the formation of compact films upon the dehydration process as a result of extensive hydrogen bonding between hydroxyl groups. This phenomenon which is known as hornification causes a reduction in features as well as functionality such as low-flux membranes, particularly for the filtration of small size or high-molecular-weight material. Surfactants, surface modification, and drying with CO_2_ can be employed to minimize this effect. Nanocellulose can also be deposited as a thin functional layer on the membrane surface.

v. Homogeneity of Nanocellulose Mixture

Another issue arising from the use of nanocellulose in composite membranes is the difficulty to obtain a homogenous mixture between cellulose and the inorganic nanoadsorbents, which will later have adverse effects on the structure, properties, and performance of the membranes.

## 6. Opportunities

In the last few years, growing interest has been shown in the utilization of agricultural waste as a raw material for nanocellulose production. Agricultural waste has become an attractive source of nanocellulose to replace the cellulosic substrate from the wood of higher plants. In fact, isolation and characterization of nanocellulose from agricultural wastes such as sugarcane and cassava bagasse, pineapple fibers, vegetable and fruit peels such as potato, carrot, tomato, and banana have been reported in a number of research papers [6,163]. This research area is particularly interesting and should be investigated further as it can minimize environmental pollution issues and is in line with the circular bioeconomy concept. In addition, the application of green technologies such as microwave- and ultrasound-assisted methods and green chemicals such as hydrogen peroxide, in nanocellulose production, is also gaining popularity among researchers due to the remarkable environmental and economic impact of the conventional multi-step isolation method utilizing concentrated alkali and acid solutions.

Despite the challenges in nanocellulose production and their applications thereof, nanocellulose offers unprecedented opportunities for tuning the membrane properties and opens a new horizon in the development of advanced multifunctional membranes. An excellent example of a multifunctional membrane based on nanocellulose is reported by Derami et al. (2020) [164]. The synergistic actions of adsorption on mesoporous polydopamine (mPDA) nanoparticles and catalytic degradation by palladium nanoparticles enabled the simultaneous removal of cationic, anionic, and neutral dyes over a wide range of concentrations, and pH, and over multiple cycles of reuse through a simple membrane filtration process. Another example is the use of Pickering emulsion as a stabilizer for nanocellulose–anatase TiO_2_ hybrid nanoparticles that were proven effective in enhancing the photocatalytic efficiency of the membrane for the simultaneous removal of water-soluble dye and organic dye. This concept is worth exploring for the development of future generation of nanocellulose-based membranes.

## Figures and Tables

**Table 1:** Density and mechanical properties of high-performance materials in comparison to nanocellulose.

Material	Density, *ρ* (g cm^−3^)	Tensile Strength, σ (GPa)	Elastic Modulus, E (GPa)	Reference
Stainless steel 304	8.00	0.50–0.70	193	[28]
E-glass fiber	2.54–2.60	0.52–3.79	72.40	[27]
TORAYCA carbon fiber	1.79	7.00	324	[30]
Kevlar 49 Aramid fiber	1.47	3.45	179	[27]
Nanocellulose	1.6	2–7.7	110–220	[26]

**Table 2:** Mechanical properties of nanocomposites reinforced with nanocellulose.

Material	Nanocellulose Composition (%)	Tensile Strength (MPa)	Tensile Strain (%)	Young’s Modulus (GPa)	Reference
GO/CNF	0	50.2 ± 6.3	4.8 ± 1.3	2.99 ± 0.31	[31]
	1	74.4 ± 2.8	4.8 ± 0.9	3.90 ± 0.84	
	4	80.0 ± 14.9	5.9 ± 2.5	4.13 ± 0.73	
SF/CNF	0	66 ± 18.9	18.6 ± 8.5	1.2 ± 0.2	[32]
	5	111.1 ± 11.7	12.7 ± 0.4	2.0 ± 0.2	
	10	140.1 ± 14.3	12.3 ± 0.6	2.7 ± 0.2	
	15	143.5 ± 8.3	11.4 ± 1.6	3.0 ± 0.1	
PNC/Nafion	0	11.5	~50	0.35	[33]
	3	15.15	~25	0.54	
	7.5	13.00	~20	0.75	
Celery CNF/Lignin/hemicellulose	0	24.1 ± 0.9	0.5 ± 0.1	4.83 ± 0.10	[34]
	10	39.0 ± 4.1	0.6 ± 0.1	7.10 ± 0.31	
	20	72.5 ± 0.3	1.1 ± 0.2	6.42 ± 0.14	
	30	79.3 ± 3.4	1.4 ± 0.1	5.62 ± 0.24	
	50	85.2 ± 2.6	2.6 ± 0.3	3.25 ± 0.16	
PAN	0	150 ± 10	44 ± 16	5.9 ± 0.4	[35]
PAN/c-CNC	0.1	190 ± 10	21 ± 5	6.7 ± 0.4	
PAN/s-CNC	0.1	190 ± 10	19 ± 6	7.0 ± 0.2	
PAN/s-CNF	0.1	150 ± 2	22 ± 7	6.3 ± 0.4	
CNC/PVA	0	117	0.7	32	[36]
	0.5	98	1.4	26	
	1.0	105	12	15	
	1.5	104	5	20	
	2.0	118	7	20	
	4.0	132	10	30	
	6.0	155	1.4	38	
CMC/CNC	0	6.10 ± 0.24	201.73 ± 0.15	na	[37]
	0.1	7.23 ± 0.71	101.05 ± 1.32		
	0.5	9.98 ± 0.55	70.53 ± 0.23		
	1	12.30 ± 0.30	89.53 ± 0.18		
PLA/LNC	0	40 ± 1	70 ± 20	1.77 ± 0.10	[38]
	1	45 ± 3	30 ± 10	1.74 ± 0.14	
	3	26 ± 1	>230	1.13 ± 0.10	
	5	27 ± 4	>130	1.06 ± 0.06	
	10	21 ± 2	35 ± 10	1.01 ± 0.06	
	20	18 ± 2	30 ± 10	0.95 ± 0.03	
PEO/CNC	0	14.2 ± 0.9	86 ± 14	0.76 ± 0.19	[39]
	1	15.9 ± 0.1	495 ± 43	0.82 ± 0.20	
	4	16.0 ± 0.8	504 ± 34	0.90 ± 0.14	
	7	17.6 ± 0.7	526 ± 40	0.94 ± 0.15	
	10	15.3 ± 0.2	416 ± 43	0.76 ± 0.33	
PEO/CNF	1	17.7 ± 0.9	491 ± 21	0.90 ± 0.10	[39]
	4	20.8 ± 0.7	281 ± 56	0.99 ± 0.22	
	7	27.3 ± 0.9	340 ± 62	1.73 ± 0.10	
	10	14.4 ± 0.5	89 ± 55	1.24 ± 0.10	

**Table 4:** Nanocellulose-based adsorbents for the removal of specific pollutants.

Membrane Material	Target Compound	Adsorption Capacity (mg/g)	Removal Efficiency (%)	Reference
Amino-modified nanocellulose	Boron	120.9	86.73	[99]
(EFB)-based nanocellulose functionalized with activated carbon	Pb^2+^	24.94	86	[101]
Electrospun CS/PEO/PNC	Cd^2+^	62.3	n.a	[70]
TOCNF/graphene oxide/trimethylolpropane-tris-(2-methyl-1-aziridine) propionate	Pb^2+^	571	n.a	[102]
	Cu^2+^	462		
	Zn^2+^	361		
	Cd^2+^	263		
	Mn^2+^	208		
TOCNF/Si/NH_2_	Cu^2+^	99.0	95.6	[103]
	Cd^2+^	124.5	85.2	
	Hg^2+^	242.1	96.9	
Magnetic grass nanocellulose	Cerium (III)	353.04	n.a	[104]
Cellulose microcrystalline for TLC	Disperse yellow	n.a	62.5	[105]
Cinnamon nanocellulose	Methyl orange	n.a	90.4 ± 2.3	[106]
Cross-linked poly(2-methacryloyloxyethyl phosphorylcholine) and bacterial nanocellulose	Methylene blue	4.44 ± 0.32	n.a	[107]
	Methyl orange	4.56 ± 0.43		
Electrospun PHA/CNC/Cs	Congo red	18.95	75.8	[108]
EDTA-embedded nanocellulose	Methylene blue	n.a	91.14	[109]
Acid-Resistant Chitosan/CNF	Methylene blue	14.71	n.a	[110]
Nanocellulose/SiO_2_	Tar	n.a	92.23	[111]
	Total particulate matter		90.25	
	Nicotine		95.02	
	CO		20.63	

**Table 5:** Nanocellulose-based photocatalysts for pollutant degradation.

Membrane Material	Degraded Compound	Photocatalytic Performance	Reference
Anatase TiO_2_/CNF	Methyl orange	99.72% degradation within 30 min, no obvious activity loss after reused for five cycles	[114]
CeO_2_/TiO_2_-CNC	Rhodamine B Methyl orange Cr(VI)	Complete removal of MO and RhB, and reduction of Cr(VI) solution within 70, 50, and 60 min	[98]
ZnO/NC	Enrofloxacin	97% degradation efficiency within 120 min	[115]
Ag_3_PO_4_/NC	Methyl orange	90% degradation efficiency in DI and 70% in wastewater within 80 min	[112]
Fe-doped ZnO/NC	Methylene blue	98.84% degradation efficiency within 90 min, 92% degradation efficiency after reused for 5 cycles	[116]
NC/γ–Fe_2_O_3_–ZrO_2_	Congo red	Increase degradation efficiency from 80.0% to 98.5% in 30 min	[117]
TiO_2_/CNC	o-chloranil	~90% degradation after 2 h	[118]
TiO_2_/CNC	Methyl orange	100% degradation in less than 6 h	[119]
CNF//PEI/Ag	Methylene blue Congo red	Up to 98% degradation efficiency after 10 times reuse, high water flux (up to 5 × 104 L·m^−2^ h^−1^)	[120]

**Table 6:** Nanocellulose-based pollutant sensors.

Type of Sensor	Material	Target Pollutant	Reference
Electrochemical sensor	d-penicillamine anchored nano-cellulose (DPA-NC) modified pencil graphite electrode	Copper ions	[124]
Colorimetric sensor	Aromatic imide functionalized nanocellulose and branched polyethyleneimine	Fluoride	[101]
Optical plasmonic chemosensor	Copper nanoparticles embedded with flexible nanocellulose	Cyanide	[126]
Biosensor	Cyanobacterial C-phycocyanin (CPC)/TOCNF	Copper ions	[127]
Optical sensor	In situ synthesized AgNPs embedded nanopaper	Chiral compounds	[128]
Optical sensor	Carbon quantum dots embedded nanopaper	Iodide	[129]
Electrochemical sensor	Rice-husk derived CNF and TOCNF/glycerol	Water soluble gases (ammonia, acetone, methane, hydrogen sulfide)	[125]
Fluorescent sensor	Carbon Dots-Rhodamine B (CDs-RhB) nanohybrid on nanopaper	Cadmium (Cd), lead (Pb), mercury (Hg), copper (Cu) and iron (Fe) ions	[130]
Optical sensor (SERS)	Gold nanorod/Silver nanocubes (AuNRs/AgNCs) embedded on bacterial nanocellulose network	2,4,6-trinitrotoluene (TNT)	[131]
Chemiresistive sensor	Nanocellulose/graphene oxide membrane attached to SnO_2_ nanosheets (NSs)	Hydrogen gas	[132]

## Data Availability

Not applicable.
